# Expression of Circulating MicroRNAs and Myokines and Interactions with Serum Osteopontin in Type 2 Diabetic Patients with Moderate and Poor Glycemic Control: A Biochemical and Molecular Study

**DOI:** 10.1155/2021/7453000

**Published:** 2021-12-07

**Authors:** Hadeel A. Al-Rawaf, Ahmad H. Alghadir, Sami A. Gabr

**Affiliations:** ^1^Department of Clinical Laboratory Sciences, College of Applied Medical Sciences, King Saud University, P.O. Box 10219, Riyadh 11433, Saudi Arabia; ^2^Department of Rehabilitation Sciences, College of Applied Medical Sciences, King Saud University, P.O. Box 10219, Riyadh 11433, Saudi Arabia

## Abstract

**Background:**

Cellular miRNAs are expressed in tissue fluids with sufficient amounts and were identified as potential molecular targets for studying the physiological mechanisms and correlations with many human diseases particularly diabetes. However, molecular-based changes among older adults with diabetes mellitus (DM) are rarely fully elucidated.

**Aim:**

This study is aimed at identifying circulating miRNAs, which hold the potential to serve as biomarkers for the immune-inflammatory changes in older T2D patients with moderate and poor glycemic control status. In addition, the association of both myokines and osteopontin (OPN) levels with circulating miRNAs was identified.

**Methods:**

A total of 80 subjects aged 20–80 years were invited during the period of October 2017–May 2018 to participate in this descriptive cross-sectional study. All subjects were diagnosed with T2D for more than 5 years. Subjects were grouped based on glycemic control (HbA1c values) into two groups: moderate glycemic control (>7-8% HbA1c, no = 30) and poor glycemic control (>8% HbA1c, no = 50), respectively. Diabetic control parameters, fasting blood sugar (FS), HbA1c, fasting insulin (IF), insulin resistance (IR), HOMA-IR, inflammatory cytokines (IL-6, IL-8, IL-18, IL-23, TNF-*α*, and CRP), osteopontin, and myokines (adropin and irisin) were estimated by colorimetric and immune ELISA assays, respectively. In addition, real-time RT-PCR analysis was performed to evaluate the expression of circulating miRNAs, miR-146a and miR-144, in the serum of all diabetic subjects.

**Results:**

In this study, T2D patients with poor glycemic control showed a significant increase in the serum levels of IL-6, IL-8, IL-18, IL-23, TNF-*α*, CRP, and OPN and a reduction in the levels of myokines, adropin and irisin, compared to patients with moderate glycemic control. The results obtained are significantly correlated with the severity of diabetes measured by HbA1c, FS, IF, and HOMA-IR. In addition, baseline expression of miR-146a is significantly reduced and miR-144 is significantly increased in T2D patients with poor glycemic control compared to those with moderate glycemic control. In all diabetic groups, the expression of miR-146a and miR-144 is significantly correlated with diabetic controls, inflammatory cytokines, myokines, and serum levels of OPN. Respective of gender, women with T2D showed more significant change in the expressed miRNAs, inflammatory cytokines, OPN, and serum myokine markers compared to men. ROC analysis identified AUC cutoff values of miR-146a, miR-144, adropin, irisin, and OPN expression levels with considerable specificity and sensitivity which recommends the potential use of adropin, irisin, and OPN as diagnostic biomarkers for diabetes with varying glycemic control status.

**Conclusion:**

In this study, molecular expression of certain microRNA species, such as miR-146a and miR-144, was identified and significantly associated with parameters of disease severity, HbA1c, inflammatory cytokines, myokines, and serum osteopontin in T2D patients with moderate and poor glycemic control. The AUC cutoff values of circulating miRNAs, miR-146a and miR-144; myokines, adropin and irisin; and serum OPN were significantly identified by ROC analysis which additionally recommends the potential use of these biomarkers, miR-146a, miR-144, adropin, irisin, and OPN, as diagnostic biomarkers with considerable specificity and sensitivity for diabetes in patients with varying glycemic control status.

## 1. Introduction

Diabetes mellitus is the most drastic disease that prevailed among older adults [1]. More than 151 million people worldwide are suffering from diabetes and its complications. The incidence rates of diabetes are expected to rise to 366 million by 2030 [1, 2]. Worldwide statistical analysis showed that diabetes significantly increased among adults by 6.4% to 7.7% [3, 4], whereas poor long-term glycemic control in patients with DM can lead to a wide range of microvascular and macrovascular complications such as renal, retinal, and primarily cardiovascular complications [4].

Type 2 diabetes mellitus (T2DM) is a progressive disease significantly associated with clinically impaired glucose regulation [5]. The progression and the development of hyperglycemia are associated with many physiological as well as cellular alterations in insulin, glucagon, and somatostatin production. In addition, the endocrine pancreas secretions and insulin action in skeletal muscle, adipose tissues, and other organs are the known hallmarks of T2DM [6–10].

Physiological changes in the cellular proinflammatory cytokines were reported in patients with diabetes which significantly play a critical role in the pathogenesis of diabetic complications via multiple biochemical and cellular pathways [11–13].

The development of diabetes and glucose disorders has epidemiologically predicted the change in inflammatory markers [14, 15], whereas a significant increase in the humoral inflammatory markers was reported in patients with type 2 diabetes [14–17]. Moreover, elevated levels of C-reactive protein (CRP), tumor necrosis factor-*α* (TNF-*α*), IL-6, IL-18, and IL-1*β* were reported among patients with type 2 diabetes mellitus displaying features of the insulin resistance syndrome [18–23].

Also, other cytokines such as osteopontin (OPN), a multifunctional protein secreted from several different cell types including bone cells and adipocytes, showed to be involved in the regulation of human glucose metabolism. Any alterations of these cytokines might be convoluted in the pathogenesis of T2DM. In patients with T2DM, OPN has been shown to be increased probably involved in subclinical inflammation [9] and insulin resistance [24].

Higher serum levels of OPN are significantly correlated with diabetic complications such as severe diabetic albuminuria and glomerulosclerosis various models of diabetic nephropathy [13, 25–27], whereas OPN was identified as a mediator involved in most chronic inflammatory and autoimmune diseases [25, 28] and subsequently demonstrated to play an important role in the diabetic-related consequences such as cardiovascular diseases [25, 29].

The correlation of cellular myokine secretion and the severity of diabetes were identified in subjects with DM type 2 [30, 31]. Previously, it was reported that both irisin and adropin expression is significantly reduced in patients with DM type 2. Moreover, a positive correlation between glycated hemoglobin (HbA1c) and both irisin and adropin was observed [30, 31]. In addition, the serum levels of irisin and adropin in patients with DM type 2 were identified to be connected to the change in metabolic factors [32, 33], such as insulin resistance index [34] and endothelium dysfunction [35–37].

Recent molecular-based studies recommended cellular miRNAs as potential targets for studying the physiological mechanisms and correlations with many human diseases [38–42]. Circulating miRNAs are short noncoded RNAs with effective posttranscriptional regulatory properties which are expressed and easily present in all biological fluids particularly, urine, saliva, serum, and plasma [38–42]. Thus, it could be used as prognostic markers for many human diseases with different pathophysiologies [43]. Recently, miRNAs were featured efficiently in association with insulin production, residual *β*-cell function, and disease complications of diabetes [44–46]. The data previously reported the potential epigenetic control of miRNAs along with other molecular targets, DNA methylation patterns, and histone modifications in the regulation of diabetes [47–49].

Although circulating miRNA levels were assessed recently in younger patients with T1D and correlated positively with the variations in cytokine levels as measures of immune-mediated signaling pathways proceed in the pathogenesis of diabetes [50–52], little is known about the potential role of miRNAs and its association with myokines and osteopontin (OPN) levels in older patients with type 2 diabetes. We postulated in this study that the expression of circulating miRNAs could reflect the immune-inflammatory effect in older patients with DM type 2. Thus, the aim of this study was to identify circulating miRNAs, which hold the potential to serve as biomarkers for the immune-inflammatory changes in older T2D diabetic patients with moderate and poor glycemic control status. In addition, the association of both myokines and osteopontin (OPN) levels with circulating miRNAs was identified.

## 2. Materials and Methods

### 2.1. Subjects

A total of 80 subjects aged 20–80 years were invited during the period of October 2017–May 2018 to participate in this descriptive cross-sectional study. Based on the American Diabetes Association criteria [53], the subjects were diagnosed with T2D for more than 5 years. Subjects with HbA1c values more than 6.5% represented noncontrolled diabetes (type 2 diabetic patients). Thus, subjects were grouped based on glycemic control (HbA1c values) into two groups: moderate glycemic control (>7-8% HbA1c, no = 30) and poor glycemic control (>8% HbA1c, no = 50), respectively. Subjects with obesity (BMI ≥ 25), type 1 diabetes, and anemia; smokers; and subjects with heart diseases and chronic diabetic complications such as nephropathy, neuropathy, retinopathy, chronic liver disease, and hypothyroidism and who use drugs (diuretics, oral contraceptives) were excluded from this study. All participants were subjected to standard anthropometric measurements to estimate BMI, WHR, and WC according to the World Health Organization [54]. The study protocol was reviewed according to the ethical guidelines of the 1975 Declaration of Helsinki and approved by the ethical committee of RRC, King Saud University, Kingdom of Saudi Arabia, under file number ID: RRC-2017-086, and signed informed consent forms were received from all subjects prior to data collection. Blood was collected from all subjects, and serum samples were obtained following centrifugation for 1 min. at 1400 rpm, were given a coded study identification number, and were shipped frozen at 20°C until reused for analysis. Demographic and clinical data of the participants are in Table 1.

### 2.2. Assessment of Glucose Control

A colorimetric assay was performed to estimate blood glucose for each participant using the QuantiChrom Glucose bioassay kit (DIGL-100, BioAssay Systems, Hayward, CA, USA). In addition, HbA1c and insulin serum levels were estimated using a commercial kit (Bio-Rad, Richmond, CA, USA) for HbA1c and immune assay ELISA kit (human insulin ELISA kit, KAQ1251, Invitrogen Corporation, Camarillo, CA, USA) for insulin, respectively. Insulin resistance in the fasting state was determined from the data of fasting insulin (IF) and fasting glucose (GF) using a validated homeostasis model assessment (HOMA-IR) as previously reported [55–57].

### 2.3. Assessment of Serum Osteopontin (OPN)

A quantitative immunosorbent assay (ELISA) technique was performed to estimate the concentrations of OPN in the serum of each subject by using a human osteoprotegerin (OPG) ELISA kit (RayBio® (OPG) ELISA Kit, catalogue #: ELH-OPG, Norcross, Georgia 30092). The procedures proceed according to the prescribed manufacturer's instructions, and the concentration of OPN was measured at 450 nm immediately by a complete set of ELISA reader model SLT Spectra 216687.

### 2.4. Assessment of Serum Cytokines

An enzyme-linked immunosorbent assay was performed to estimate the levels of IL-6, IL-8, IL-18, IL-23, TNF-*α*, and CRP by using a Quantikine Human Immunoassay ELISA kit (R&D System, Minneapolis, USA). The procedures were run according to an accurate ELISA manufacturer's protocol. A standard curve was used to determine cytokine levels, and the concentration of each cytokine was expressed as pg/mL.

### 2.5. Assessment of Serum Myokines

Serum adropin and irisin levels were estimated by immune assay technique as previously reported [58] by using commercially available kits (human adropin ELISA kit, catalogue no. CK-e90267, Hangzhou Eastbiopharm Co., Blue Ocean International Times Mansion, China, for adropin and human irisin ELISA kit, catalogue no. CK-E90905, Hangzhou Eastbiopharm Co., Blue Ocean International Times Mansion, China, for irisin), respectively [58]. The sensitivity limits of the assays are 2.49 ng/L for adropin and 0.023 *μ*g/mL for irisin with interassay coefficients of variance < 10% and <12%, respectively. In addition, the detection range of adropin was 5-1000 ng/L and for irisin was 0.05-15 *μ*g/mL [58].

### 2.6. Real-Time RT-PCR Analysis of Circulating miRNAs and Apoptotic Genes

#### 2.6.1. Extraction of RNA and Synthesis of *cDNA*

For each participant, the miRNeasy isolation kit (Qiagen, Hilden, Germany) was used to extract total RNA from serum samples. A reverse transcription polymerase chain reaction (RT-PCR) was applied to analyze total RNA in all serum samples. Then, a complementary DNA (cDNA) was generated using reverse transcription miScriptII RT kits (Qiagen), and the levels of miRNAs were evaluated by optical density [4, 5].

#### 2.6.2. Real-Time RT-PCR Analysis

The primers of circulating miRNAs, miR-146a and miR-144 (Applied Biosystems, Foster City, CA, U.S.A.), were used to screen the expression of miRNAs in the plasma of all participants by using quantitative real-time RT-PCR [38]. The average copy number of the resultant PCR components was normalized according to the GAPDH gene which was used as an internal housekeeping gene [59]. In the PCR process, templates of respective cDNA were subjected to four thermal phases: primary denaturation phase (I) (at 94°C for 2 minutes), denaturation phase (II) (at 94°C for 30 seconds), annealing phase (III) (at 59°C for 30 seconds), and amplification phase (IV) (at 72°C for 30 seconds). The PCR phases (II to IV) proceed for 45 cycles, and all reactions were measured in a triplicated manner [59].

#### 2.6.3. Statistical Analysis

Power calculations of the selected sample size of 80 subjects showed to give an estimated power of 95% and a significance level of 0.05 with an expected frequency of 11.6%.

An SPSS statistical program (SPSS, IBM Statistics V.17) was used to analyze all data produced in this study. The data of continuous variables are expressed as the mean ± SD. A nonparametric test (Mann-Whitney-Wilcoxon test) and the *χ*^2^ test were used to analyze the frequency of the differences between the studied groups, respectively.

Two independent sample *t*-tests were used for comparison between the studied variables such as diabetes (dependent variable) and expression levels of miRNAs, cytokines, myokines, and serum osteopontin (independent variables). In addition, multiple stepwise regressions and Pearson's correlation analysis were used to estimate the associations between diabetes and the studied independent variables in patients with type 2 diabetes. The correlation coefficient was translated into descriptors like “weak,” “moderate,” or “strong” relationship. It is mostly agreed that a coefficient of <0.1 indicates a negligible and >0.9 a very strong relationship, whereas values inbetween are differential [60, 61]. The strength of correlation in our study ranged as moderate correlation (0.40–0.69) and strong correlation (0.70–0.89). The susceptibility and sensitivity of myokines, osteopontin, and expressed miRNAs for diagnosis of diabetes at baseline expression were determined using the area under the receiver operating characteristic (ROC) curve as previously reported [39]. All tests were two-tailed; because of multiple assessments, results were only considered statistically significant at a value of *p* < 0.05.

## 3. Results

In this study, the analysis was performed on a total of 80 serum specimens (58.75% male vs. 41.25 female). Based on HbA1c controls, the subjects were grouped into two groups: moderate glycemic controlled (>7-8% HbA1c, no = 30) and poor glycemic controlled (>8% HbA1c, no = 50) type 2 diabetes (T2D) subjects, respectively (Table 1). The results showed no significant difference in the adiposity measures: fat-free mass, fat mass, and BMI in diabetic patients compared with healthy controls. In addition, it was found that the mean levels of diabetic parameters, FBG, HbA1c, FINS, and HOMA-IR, are significantly elevated in poor glycemic controlled T2D patients compared to moderate glycemic controlled T2D patients (Table 1).

**Figure 1:**
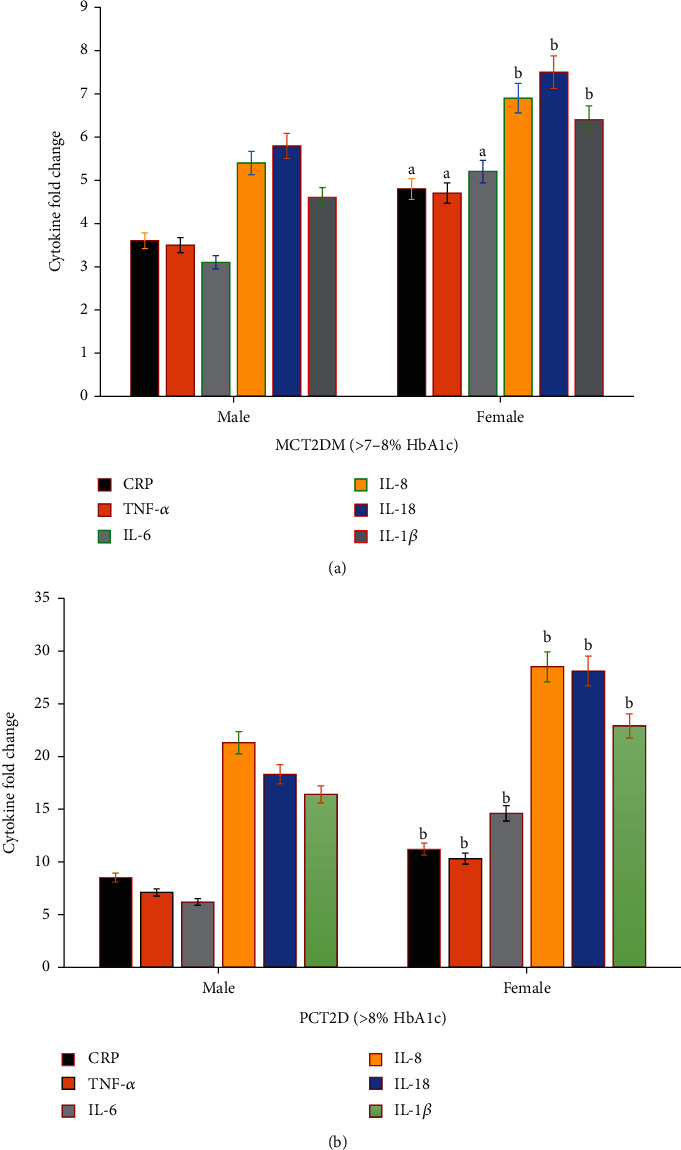
Gender differences in fold changes of proinflammatory cytokines (CRP, TNF-*α*, IL-6, IL-8, IL-18, and IL-1*β*) in moderate glycemic controlled T2D (MCT2D; >7-8% HbA1c, no = 30) (a) and poor glycemic controlled T2D (PCT2D; >8% HbA1c, no = 50) (b). Females of controlled and poor type 2 diabetic groups showed significant increase in all studied cytokines compared to males of the same group. Significance based on gender difference (male vs. female) at ^a^*p* < 0.01 and ^b^*p* < 0.001.

In this study, in order to explore the potential role of the parameters of glucose control in cellular inflammation, the levels of proinflammatory cytokines and myokines were estimated and compared based on diabetes status (Table 2). In individuals with poor glycemic controlled T2D, significantly high levels of IL-6, IL-8, IL-18, IL-1*β*, and TNF-*α* were evaluated compared to moderate glycemic controlled T2D patients (*p* = <0.01). In addition, serum CRP levels significantly increased (*p* = <0.001) in poorly controlled T2D compared to moderately controlled T2D patients (HbA1c > 7-8%) (Table 2). Additionally, when proinflammatory cytokines were compared by levels of HbA1c, we observed that IL-6, IL-8, IL-18, IL-1*β*, TNF-*α*, and serum CRP levels were significantly elevated in poorly controlled T2D with higher HbA1c values compared to moderately controlled T2D patients (Table 2). According to gender difference, a change in cytokines was reported also as shown in Figure 1. Females of the moderately controlled T2D patient (Figure 1(a)) and poorly controlled T2D (Figure 1(b)) groups showed a significant increase (*p* = 0.001) in all studied cytokines compared to males of the same group.

**Figure 2:**
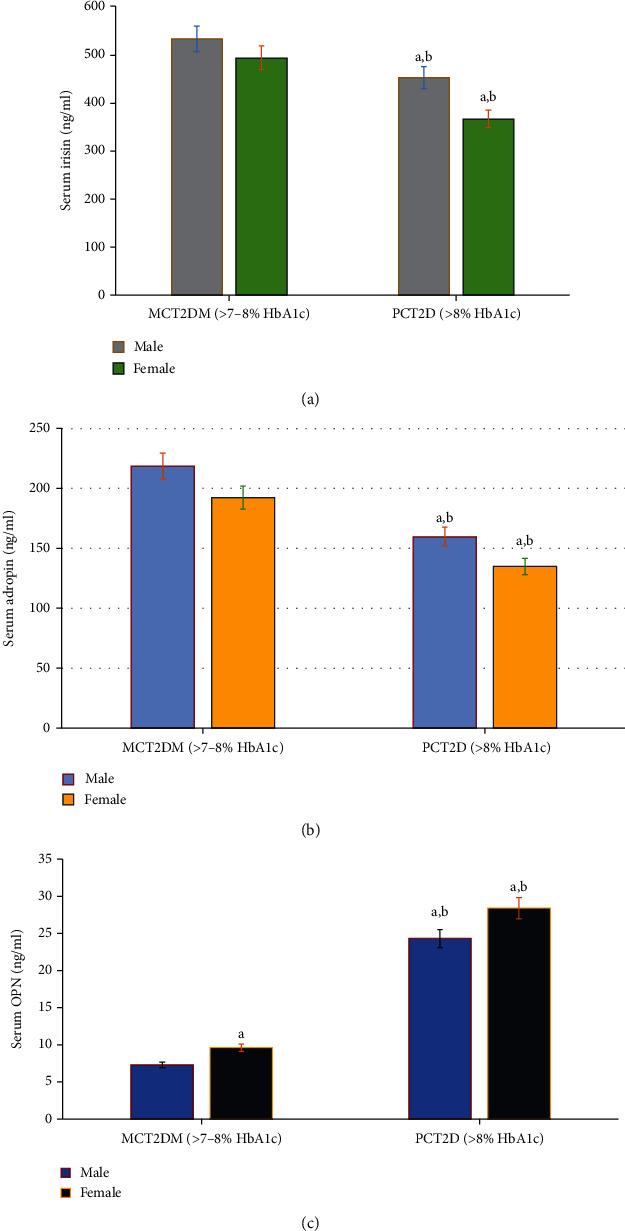
Changes in osteopontin (OPN) (a) and myokines, irisin (b) and adropin (c), in moderate glycemic controlled T2D (MCT2D; >7-8% HbA1c, no = 30) and poor glycemic controlled T2D (PCT2D; >8% HbA1c, no = 50). The results showed significant increase (*p* = 0.001) in the levels of OPN (a) and decrease (*p* = 0.001) in the levels of irisin (b) and adropin (c) in poor glycemic controlled T2D patients compared to moderate glycemic controlled T2D. In addition, female patients showed higher significant change (*p* = 0.001) in the studied parameters (OPN, irisin, and adropin), respectively, compared to male subjects of the same group. Significance at ^a^*p* < 0.01 (male vs. female); ^b^*p* < 0.001 (moderately controlled vs. poorly controlled type 2 diabetes).

Interestingly, participants with poorly controlled T2D tended to have lower levels of irisin and adropin compared to moderately controlled T2D patients. Both serum levels of adropin and irisin were significantly (*p* = 0.001) reduced in patients with poorly controlled T2D compared with moderately controlled T2D patients (Table 2 and Figures 2(a) and 2(b)). These differences remained significant when compared on the basis of HbA1c levels, suggesting an important role of these myokines in both moderately and poorly controlled diabetes. The results also showed that female patients have recorded a higher significant change (*p* = 0.001) in the studied myokines, irisin and adropin, respectively, compared to male subjects of the same group as shown in Figures 2(a) and 2(b).

In this study, serum OPN was measured in the serum of both controlled nondiabetic subjects and type 2 diabetic patients (Figure 2(a)). The serum levels of OPN significantly increased in poorly controlled T2D compared to moderately controlled T2D patients (Figure 2(c)). Based on gender difference, serum OPN was significantly elevated in females with poorly controlled T2D compared to males of the same group. In moderately controlled T2D patients, although females showed higher serum OPN levels than males, the results are statistically nonsignificant (Figure 2(c)). In addition, the expressed OPN significantly moderately to strongly correlated with HbA1c, FINS (mUI/mL), and HOMA-IR and cytokines IL-6, IL-8, IL-18, IL-1*β*, TNF-*α*, and serum CRP levels, respectively, in patients with poorly controlled T2D compared to moderately controlled T2D patients (Table 3).

Serum myokines showed to be associated with BMI, HbA1c levels, increased levels of cytokines, and serum OPN levels in patients with moderate and poorly controlled T2D. The expression of both adropin and irisin is significantly correlated positively with HbA1c levels and negatively with BMI, serum OPN, and cytokines IL-6, IL-8, IL-18, IL-1*β*, TNF-*α*, and serum CRP levels, respectively, as shown in Table 4. In patients with poorly controlled T2D, the strength of the correlation of both adropin and irisin ranged between moderate to strong compared to that moderate correlation present in patients with moderately controlled T2D, respectively (Table 4).

**Figure 3:**
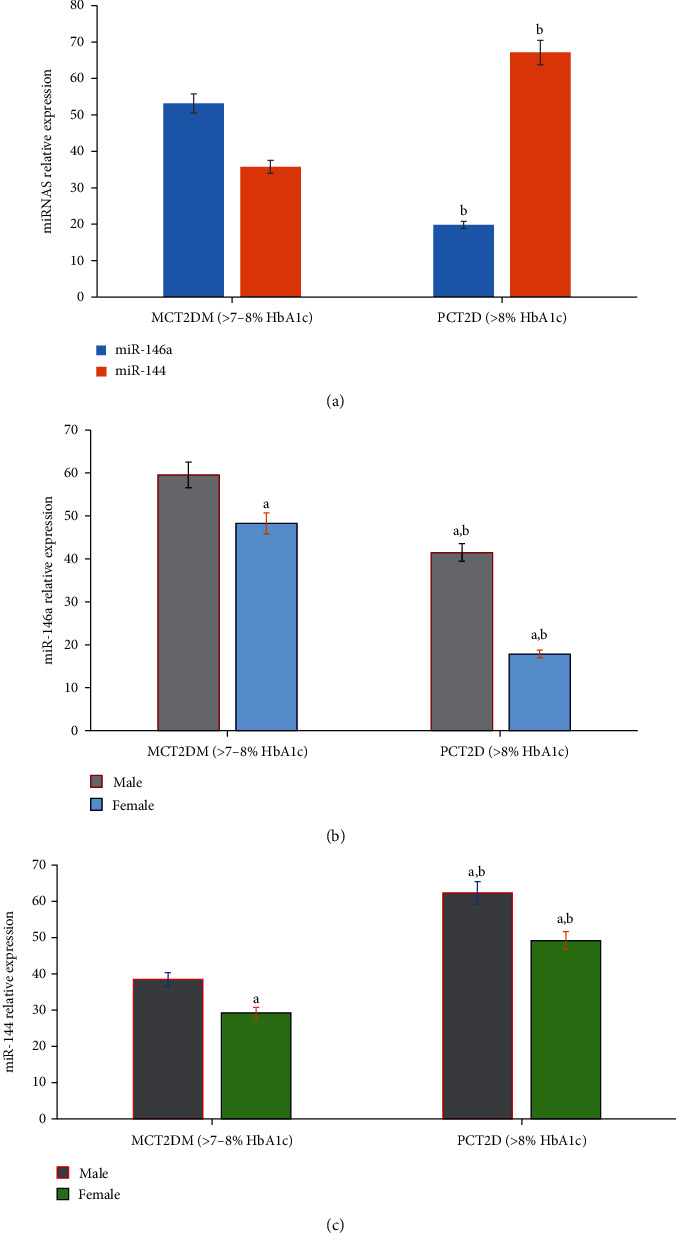
MicroRNAs' differential expression profile in moderate glycemic controlled T2D (MCT2D; >7-8% HbA1c, no = 30) and poor glycemic controlled T2D (PCT2D; >8% HbA1c, no = 50) (a). The results showed that the relative expression of miR-146a significantly decreased (*p* = 0.001) and miR-144 significantly increased (*p* = 0.001) in poor glycemic controlled T2D patients compared to moderate glycemic controlled T2D. In addition, female patients showed more significant change in the expression levels of miR-146a (b) and miR-144 (c) compared to that of male subjects of the same group. Significance at ^a^*p* < 0.01 (male vs. female); ^b^*p* < 0.001 (moderately controlled vs. poorly controlled type 2 diabetes).

The potential role of cellular miRNAs in the pathogenesis of diabetes and its correlation with cytokines and myokines were evaluated in this study by real-time PCR analysis. The expression of miR-146a and miR-144 was estimated in the serum of both moderate and poor glycemic controlled patients as shown in Figure 3. Interestingly, the level of miR-146a and miR-144 was markedly changed (*p* = 0.001) in the serum of poor glycemic controlled patients compared to moderate glycemic controlled patients. The levels of miR-146a significantly decreased, and the levels of miR-144 significantly increased in poor glycemic controlled patients compared to moderate glycemic controlled patients (Figure 3(a)). In addition, a more significant change in the expression levels of miR-146a (Figure 3(b)) and miR-144 (Figure 3(c)) was reported in women compared to men subjects of the same group.

The expression of miR-146a and miR-144 in the serum of T2D patients with moderate and poor glycemic control status significantly was correlated with serum levels of OPN, cytokines, and myokines as well as HbA1c, FINS (mUI/mL), and HOMA-IR levels, respectively (Table 5). Both miR-146a and miR-144 correlated positively with myokines, adropin and irisin, and negatively with HbA1c levels, OPN, and cytokines IL-6, IL-8, IL-18, IL-1*β*, TNF-*α*, and serum CRP levels, respectively, as shown in Table 5. The strength of the correlation significantly ranged from moderate to strong (*R*) in patients with poor glycemic control compared to that present in patients of moderately controlled HbA1c levels which showed moderate rates of correlation strengths (Table 5).

In addition, ROC analysis was performed to explore the potential use of miR-146a, miR-144, OPN, adropin, and irisin expression levels as diagnostic biomarkers for both moderate and poor glycemic controlled patients as shown in Tables 6 and 7. In T2D patients with moderate glycemic control, the data showed that the AUC was 0.81 (0.72-0.98) for miRNA-146a, with a sensitivity of 91.5% and specificity of 89.4%, and for miRNA-144, AUC was 0.78 (0.69-086), with a sensitivity of 84.7% and specificity of 86.8% at best cutoff values as shown in Table 6, which indicates that the miR-146a and miR-144 levels were strong diagnosis biomarkers of diabetes with different glycemic controlled status. Also, the results showed that the AUC cutoff value of adropin was 0.82 (0.73-0.93) with a sensitivity of 79.1% and specificity of 71.6%; for irisin, AUC was 0.78 (0.65-0.89), with a sensitivity of 76.2% and specificity of 72.3%; and for OPN, AUC was 0.91 (0.75-0.95), with a sensitivity of 88.6% and specificity of 75.1% (Table 6).

Similarly, in T2D patients with poor glycemic control, ROC analysis showed variable values of the studied biomarkers and their correlation with the severity of the T2D. The AUC analysis was 0.91 (0.72-0.98) for miRNA-146a, with a sensitivity of 94.5% and specificity of 91.4%, and for miRNA-144, AUC was 0.86 (0.69-0.93), with a sensitivity of 88.7% and specificity of 87.8% at best cutoff values as shown in Table 7; these cutoff values significantly indicate that the miR-146a and miR-144 levels will be a strong diagnosis biomarker of diabetes with poor glycemic controlled status. Also, the results showed that the AUC cutoff value was 0.87 (0.73-0.93) for adropin with a sensitivity of 82.1% and specificity of 79.6%; for irisin, AUC was 0.82 (0.65-0.91), with a sensitivity of 79.4% and specificity of 76.5%; and for OPN, AUC was 0.96 (0.75-0.98), with a sensitivity of 89.7% and specificity of 79.3% (Table 7).

The strategy of ROC analysis was used to diagnose diabetes in adult patients with moderate and poor glycemic control status. In T2D patients with moderate glycemic control status, the current circulating miRNAs, miR-146a and miR-144; myokines, adropin and irisin; and serum OPN yielded a range of 76.2-91.5% specificity and 71.6–89.4% sensitivity. In T2D patients with poor glycemic control, the studied biomarkers provide a good range of 79.4-94.5% specificity and 76.5–91.4% sensitivity. These best cutoff values additionally recommend the potential use of adropin, irisin, and OPN as diagnostic biomarkers for diabetes with varying glycemic control status.

## 4. Discussion

In this study, circulating miRNA levels in serum samples from adult patients with T2D with moderate and poor glycemic control status are significantly associated with the parameters of diabetic controls, HbA1c, FINS, and HOMA-IR, respectively. In the present study, two miRNAs, miR-146a and miR-144, were identified with expression levels that were influenced by disease progression and increased serum levels of inflammatory cytokines, myokines, and osteopontin (OPN). In recent studies, the role of miRNAs was explored and showed to be associated with the pathogenesis of diabetes mellitus in all ages [45, 51, 62, 63], whereas a profound impairment of glucose metabolism [64] was reported in association with a dysregulation in the expression of miRNAs.

In the same study population, serum levels of inflammatory cytokines, IL-6, IL-8, IL-18, IL-1*β*, and TNF-*α* and serum CRP levels were associated with the progression of the T2D disease. IL-6, IL-8, IL-18, IL-1*β*, TNF-*α*, and CRP levels were significantly increased in T2D patients with poor glycemic control (>8% HbA1c) compared to patients with moderate glycemic control (>7-8% HbA1c). Respective of gender, the levels of inflammatory cytokines significantly increased in females with type 2 diabetes compared to males of the same group.

Previously, a state of the innate immune system was represented in type 2 diabetes mellitus which significantly leads to an increase in the response of cytokine-mediated acute phase [65]. Thus, more systemic inflammatory circulating markers such as IL-6, TNF-*α*, and CRP levels significantly increased and were detrimentally respective to the risk and the development of type 2 diabetes mellitus. In addition, these inflammatory circulating markers significantly increased in patients with featured insulin resistance and those who were clinically with overt type 2 diabetes mellitus [18–22]. Also, inflammatory such as IL-6 and TNF-*α* are significantly correlated with HbA1c and diabetic nephropathy [20–22].

In patients with hyperglycemic spikes, increased secretion levels of cytokines such as IL-6 and TNF-*α* might be associated with increased vascular risk in patients with T2D [65–68]. In a previous study of meta-analysis, T2DM risk showed to be strongly linked with elevated serum levels of inflammatory cytokines such as IL-1b, IL-6, IL-18, CRP, and TNF-*α*. Also, chronically elevated levels of inflammatory cytokines such as CRP, TNF-a, IL-6, and IL-1b could enhance insulin resistance (IR), disrupt insulin sensitivity, and consequently impair glucose homeostasis resulting in an increase in the risk of T2DM [69–75].

In our study, compared to control nondiabetic subjects, increased inflammatory cytokines were predictors of the onset of T2DM in both men and women, whereas differences in the diabetes risk between sexes depend mainly on the change in the expression levels of cytokines [76, 77]. Like our results, a significant increase in inflammatory cytokines was also reported in women with T2DM compared to men of the same disease category [78, 79]. Previous studies based upon cultured cells and animal models suggested that expressed inflammatory cytokines like IL-6, TNF-*α*, IL-1b, IL-18, and CRP significantly contributed to the pathogenesis of T2DM through interfering with the insulin signal and impairing *β*-cell function [80] and action on peripheral insulin resistance [81] as well as insulin secretion [82].

Osteopontin (OPN) is a multifunctional protein significantly expressed in different biological cell types including bone cells [83]. It was shown to be associated with several physiological and pathological conditions such as cancer, chronic inflammatory disorders, autoimmune diseases [84], and insulin resistance [85]. Moreover, OPN also had a biological role in vascular remodeling and calcification processes, particularly in diabetic arteries [86, 87], and has shown to be linked with complications of type 2 diabetes (T2D) such as retinopathy [88] and nephropathy [89].

Thus, in this study, serum OPN levels were measured in the serum of T2D patients with both moderate and poor glycemic control status. The results showed that serum levels of OPN significantly increased in patients with poor glycemic control compared to that of moderate glycemic control, respectively. However, serum OPN is highly expressed in the serum of women with T2D compared to men of the same group. In poor glycemic control patients, although females showed higher serum OPN levels than males, the results are statistically nonsignificant. In addition, increased OPN serum levels are significantly correlated with HbA1c, FINS (mUI/mL), and HOMA-IR and cytokines IL-6, IL-8, IL-18, IL-1*β*, TNF-*α*, and serum CRP levels, respectively, in T2D patients with poor compared to that of moderate glycemic control status. Previous studies reported an increase in the serum levels of OPN in diabetic patients compared to controls, which significantly contributed and was highly induced by elevated glucose and HbA1c levels [90]. The increased levels of OPN showed to be associated with severe diabetic complications such as accelerated atherosclerosis among diabetes subjects [87].

Additionally, OPN with other reported inflammatory cytokines particularly IL-6, IL-8, IL-18, IL-1*β*, TNF-*α*, and serum CRP have been implicated in hypertension associated with diabetes via mediating the vascular effects of both angiotensin II (Ang II) and aldosterone, respectively [91]. In many acute and chronic vascular or endothelial responses, OPN is a pleiotropic cytokine commonly associated with vascular damage, inflammation, and/or fibrosis induced in diabetic patients [91–93].

New muscle-secreted cytokines particularly irisin and adropin referred to as myokines are able to regulate glucose, lipid levels, insulin sensitivity, and low-grade inflammation [94–96]. The expressed myokines play a significant role in regulating metabolism and chronic inflammation via interaction with human organs such as muscle, liver, adipose tissue, and brain with various effects [97].

In our diabetic patients, the levels of both adropin and irisin are significantly (*p* = 0.001) reduced in T2D patients with poor compared to that of moderate glycemic control status. Also, a higher significant change (*p* = 0.001) in irisin and adropin was reported in women compared to men with diabetes.

Irisin and adropin are highly expressed in adipose tissue, cardiac muscle, and the heart. It showed to improve glucose homeostasis, insulin sensitivity, and weight loss via thermogenic action. It was reported to increase energy expenditure rates by transforming white adipose tissue to brown adipose tissue and regulate carbohydrate metabolism [37, 97–100]. In both T2D patients with poor and moderate glycemic control status, the results also showed that both adropin and irisin are closely correlated with HbA1c, FINS (mUI/mL), and HOMA-IR and serum cytokines. The expression of both adropin and irisin is correlated positively with HbA1c levels and negatively with BMI, serum OPN, and cytokines IL-6, IL-8, IL-18, IL-1*β*, TNF-*α*, and serum CRP levels, respectively.

In patients with D2M diabetes, irisin showed to play a significant role in insulin sensitivity and metabolic disorders [101, 102]. Significantly lower levels of irisin were reported in association with BMI, HOMA-IR, and fasting insulin [103, 104]. Moreover, a lower level of adropin is positively correlated with glycated hemoglobin (HbA1c) in patients with diabetes [30–32, 105, 106]. In elderly patients, increased levels of irisin showed to be associated with a reduced risk of diabetes mellitus type 2 and its subsequent complications such as hypertension and obesity [107].

An inverse correlation between irisin, adropin, and other related inflammatory cytokines was reported in our patients which significantly increased the pathogenicity of type 2 diabetes. Several studies reported that improving irisin and adropin levels significantly enhances glucose homeostasis and insulin resistance and reduced both inflammatory cytokine production and weight loss [97, 108–110]. The increase in the levels of irisin and adropin increases energy expenditure rates which reduces aggravated fat accumulation and obesity and enhances insulin resistance and inflammation in diabetic patients [108].

Molecular-based studies reported the significant role of circulating miRNAs as short, noncoding RNA molecular candidates which are associated with many physiological, biochemical, and pathological processes in human bodies [111–113]. Dysregulation of circulating miRNAs showed to be linked with the pathogenesis of diabetes mellitus [114], which can lead to profound impairment of glucose metabolism [62, 114]. Recently, miRNA expression profiles in serum, plasma, and urine as well as various tissues particularly the pancreas, adipose tissue, and liver from patients with T2DM have been established which gives the accessibility to discover novel miRNA regulators in diabetes [62, 64, 115, 116]. In addition, in most studies, microRNA showed to play a potential role in the severity and most diabetic complications associated with diabetes such as the inflammatory processes of atherosclerosis [117–120]. miR-342-5p, for example, has been reported to be linked with macrophage activation during atherosclerosis and cytokine secretion in CAD patients. The levels of miR-342-5p were estimated to be highly expressed and positively correlated with inflammatory cytokines [117].

The data previously reported the potential epigenetic control of miRNAs along with other molecular targets, DNA methylation patterns, and histone modifications in the regulation of diabetes [47–49]. Little is known about the potential role of miRNAs and their association with myokines and osteopontin (OPN) levels in older patients with type 2 diabetes.

In this current study, the potential role of circulating miRNAs, miR-146a and miR-144, was evaluated by using real-time PCR analysis. Significant changes were reported in the levels of miR-146a and miR-144 in diabetic patients with varying glycemic control status. The levels of miR-146a significantly decreased and the levels of miR-144 significantly increased in T2D patients with poor glycemic control status compared to those with moderate glycemic control status. Respective of gender specificity, women patients showed a more significant change in the expression levels of miR-146a and miR-144 compared to the men subjects of the same group. In addition, our results for both T2D diabetic groups showed that expressed levels of miR-146a and miR-144 correlated positively with myokines, adropin and irisin, and negatively with HbA1c levels, OPN, and cytokines IL-6, IL-8, IL-18, IL-1*β*, TNF-*α*, and serum CRP levels, respectively. Alternation of the expression levels of many miRNAs was reported in diabetic patients which significantly involved in angiogenesis, vascular repair, and endothelial homeostasis [121–123].

The expression of the miRNA-146 family showed in previous studies [138, 139] to participate with the regulation of oxidative stress and the production of proinflammatory cytokines. In addition to that, circulating levels of miR-146a are reduced in diabetic patients which significantly downregulates the expression of NADPH oxidase [124–126] and reduces nuclear factor- (NF-) *κ*B signaling in endothelial cells exposed to glucose oscillations [127, 128]. Thus, overexpression of one of the miRNA-146 families (miRNA-146a or miRNA-146b) significantly prevents increased oxidative stress and reduces the production of inflammatory factors in diabetic rodents [124, 125].

Similarly, expression of miR-144 significantly increased in patients with higher glucose levels which was associated with apoptosis in human endothelial cells whereas diabetes induced endothelial dysfunction via the apoptotic cell mechanism [129, 130].

Like other miRNAs, our identified miR-146a and miR-144 are significantly associated with inflammatory cytokines, OPN, and severity of diabetes through the apoptotic mechanism [125–128]. It was reported previously that the inflammatory process by cytokines such as TNF-*α* and IL-1*β* is responsible for recruiting T lymphocytes in response to an inflammatory process which mediates the apoptotic death of pancreatic *β* cells through the activation of T lymphocytes [131, 132]. The proposed link between expressed miR-146a and miR-144 and the inflammatory cytokines could suggest that the possible link between these miRNAs and the pathogenesis of diabetes proceed via apoptosis [133, 134].

Previous studies confirm the association of miR-146a and miR-144 in the pathogenesis of many diseases via inflammatory pathways. It showed to play a negative role in the regulation of NF-*κ*B activity via targeting the expression of many cytokines such as TNF receptor-associated factor IL-6 and IL-1 receptor-associated kinase and regulating many genes such as FAF1, IRAK2, FADD, IRF-5, Stat1, and PTC-1 that are key components of cytokine signaling pathways and inflammation in many diseases [51, 135, 136].

In previous studies, baseline levels of several miRNAs which are significantly correlated with HOMA-IR, *β*-cell function (HOMA-B) values, adiposity, inflammation, cytokines, and insulin resistance could be used as parameters to classify normoglycemic individuals who developed T2D over 10 years [123, 137–142]. Thus, the assessment of miRNA signatures along with other parameters such as myokines and serum osteopontin can help in not only accurately classifying DM but also distinguishing DM from other diseases.

In this study, ROC analysis additionally was performed to explore the potential use of miR-146a, miR-144, adropin, irisin, and OPN expression levels as diagnostic biomarkers for both moderate and poor glycemic controlled patients. The AUC values, 0.81, 0.78, 0.82, 0.78, and 0.91, respectively, with a range of specificity (76.2-91.5%) and sensitivity (71.6–89.4%) were identified. In addition, AUC values of the same biomarkers, 0.91, 0.86, 0.87, 0.82, and 0.96, respectively, with a range of specificity (79.4-94.5%) and sensitivity (76.5–91.4%) were identified in T2D patients with poor glycemic control. These best cutoff values additionally recommend the potential use of adropin, irisin, and OPN as diagnostic biomarkers for diabetes with varying glycemic control status.

## 5. Conclusion

In this study, molecular expression of certain microRNA species, such as miR-146a and miR-144, was identified and significantly associated with the parameters of disease severity, HbA1c, inflammatory cytokines, myokines, and serum osteopontin in T2D patients with moderate and poor glycemic control. The AUC cutoff values of circulating miRNAs, miR-146a and miR-144; myokines, adropin and irisin; and serum OPN were significantly identified by ROC analysis which additionally recommends the potential use of these biomarkers: miR-146a, miR-144, adropin, irisin, and OPN as diagnostic biomarkers with considerable specificity and sensitivity for diabetes in patients with varying glycemic control status.

## Figures and Tables

**Table 1:** The demographics, clinical, and metabolic characteristics of the participants.

Variables	Type 2 diabetes (no = 80)	
	Moderate glycemic control (>7-8% HbA1c, no = 30)	Poor glycemic control (>8% HbA1c, no = 50)
Genders (male/female)	18/12	29/21
Age (years)	54.6 ± 2.9	53.9 ± 2.6
BMI (kg/m^2^)	22.8 ± 2.3	22.6 ± 2.8
Fat-free mass (kg)	64.4 ± 2.4	72.4 ± 2.9
Fat mass (kg)	29.3 ± 2.3	35.1 ± 2.6
Fasting plasma glucose (mmol L^−1^)	10.8 ± 0.86	16.7 ± 0.98^b^
HbA1c (%)	7.82 ± 0.5	9.65 ± 0.9^c^
FINS (mUI/mL)	9.8 ± 2.5	14.3 ± 3.7^c^
HOMA-IR	3.1 ± 2.7	7.4 ± 3.5^b^
Diabetes duration (years)	6.5 ± 1.6	6.4 ± 1.7

Values are expressed as mean ± SD; significance at ^a^*p* < 0.05, ^b^*p* < 0.01, and ^c^*p* < 0.001. Abbreviations: BMI: body mass index; HOMA: homeostatic model assessment; IR: insulin resistance; FINS: fasting serum insulin; HbA1c: glycated hemoglobin A1c.

**Table 2:** Comparison of cytokine/myokine profiles in the serum of all subjects based on diabetes status.

Variables	Type 2 diabetes (*n* = 80)	
	Moderate glycemic controlled (>7-8% HbA1c, no = 30)	Poor glycemic controlled (>8% HbA1c, no = 50)
CRP (mg/L)	8.1 ± 2.3	11.6 ± 1.9^c^
TNF-*α* (pg/mL)	4.7 ± 1.8	8.6 ± 2.6^b^
IL-6 (pg/mL)	5.8 ± 4.1	8.2 ± 3.8^b^
IL-8 (pg/mL)	19.7 ± 5.2	27.1 ± 6.1^b^
IL-18 (pg/mL)	16.9 ± 3.6	20.5 ± 4.1^b^
IL-1*β* (pg/mL)	18.2 ± 4.5	26.1 ± 6.1^b^
Adropin (ng/mL)	141.7 ± 6.5	128.4 ± 7.2^c^
Irisin (ng/mL)	261.7 ± 3.7	196.4 ± 5.8^c^

Values are expressed as mean ± SD; significance at ^a^*p* < 0.05,^.b^*p* < 0.01, and ^c^*p* < 0.001. HbA1c: glycated hemoglobin A1c.

**Table 3:** Correlations of osteopontin (OPN) with inflammatory cytokines and clinical parameters of diabetes with different controls.

Studied parameters	OPN (*R*)	
	Moderate glycemic controlled (>7-8% HbA1c, no = 30)	Poor glycemic controlled (>8% HbA1c, no = 50)
HbA1c (%)	0.58^a^	0.68^b^
FINS (mUI/mL)	0.46^a^	0.72^b^
HOMA-IR	0.41^a^	0.46^b^
CRP (mg/L)	0.62^a^	0.78^b^
TNF-*α* (pg/mL)	0.43^a^	0.74^b^
IL-6 (pg/mL)	0.48^a^	0.59^b^
IL-8 (pg/mL)	0.68^a^	0.74^b^
IL-18 (pg/mL)	0.56^a^	0.58^b^
IL-1*β* (pg/mL)	0.46^a^	0.49^b^
Interpretation of the correlation coefficient (*R*)	Moderate *R*	Moderate-strong *R*

Data are presented as Pearson's (*R*) coefficients adjusting for variables identified as cofounders in univariate analyses. Moderate correlation coefficient (0.40-0.69); strong correlation coefficient (0.70-0.89). Significance at *p* < 0.05. ^a^*p* < 0.01; ^b^*p* < 0.001. HbA1c: glycated hemoglobin A1c.

**Table 4:** Correlations of adropin and irisin with clinical and laboratory parameters of diabetic patients with different controls.

Studied parameters	Myokines (*R*)			
	Moderate glycemic controlled T2D (MCT2D; >7-8% HbA1c, no = 30)		Poor glycemic controlled T2D (PCT2D; >8% HbA1c, no = 50)	
	Adropin	Irisin	Adropin	Irisin
BMI	-0.46^b^	-0.54^b^	-0.48^b^	-0.54^b^
HbA1c (%)	0.41^b^	0.43^b^	0.45^b^	0.43^b^
FINS (mUI/mL)	0.56^b^	0.48^b^	0.66^b^	0.48^b^
HOMA-IR	0.48^b^	0.53^b^	0.51^b^	0.53^b^
OPN	-0.45^b^	-0.65^b^	-0.55^b^	-0.65^b^
CRP (mg/L)	-0.44^b^	-0.41^b^	-0.46^b^	-0.41^b^
TNF-*α* (pg/mL)	-0.67^a^	-0.65^b^	-0.83^a^	-0.85^b^
IL-6 (pg/mL)	-0.46^a^	-0.63^b^	-0.63^a^	-0.63^b^
IL-8 (pg/mL)	-0.63^b^	-0.68^b^	-0.68^b^	-0.78^b^
IL-18 (pg/mL)	-0.42^a^	-0.45^b^	-0.52^a^	-0.45^b^
IL-1*β* (pg/mL)	-0.61^a^	-0.64^a^	-0.81^a^	-0.64^a^
Interpretation of the correlation coefficient (*R*)	Moderate *R*	Moderate *R*	Moderate-strong *R*	Moderate-strong *R*

Data are presented as Pearson's (*R*) coefficients adjusting for variables identified as cofounders in univariate analyses. Moderate correlation coefficient (0.40-0.69); strong correlation coefficient (0.70-0.89). Significance at *p* < 0.05. ^a^*p* < 0.01; ^b^*p* < 0.001. HbA1c: glycated hemoglobin A1c.

**Table 5:** Correlations of miRNAs, miR-146a and miR-144, with clinical and laboratory parameters of diabetic patients with different controls.

Studied parameters	miRNA expression			
	Moderate glycemic controlled T2D (MCT2D; >7-8% HbA1c, no = 30)		Poor glycemic controlled T2D (PCT2D; >8% HbA1c, no = 50)	
	miR-146a	miR-144	miR-146a	miR-144
HbA1c (%)	-0.41^b^	0.46^b^	-0.46^b^	0.52^b^
FINS (mUI/mL)	-0.46^b^	0.53^b^	-0.42^b^	0.58^b^
HOMA-IR	-0.58^b^	0.48^b^	-0.53^b^	0.51^b^
OPN	-0.57^b^	0.49^b^	-0.55^b^	0.79^b^
CRP (mg/L)	-0.46^b^	-0.52^b^	-0.47^b^	-0.54^b^
TNF-*α* (pg/mL)	-0.54^a^	-0.85^b^	-0.59^a^	-0.87^b^
IL-6 (pg/mL)	-0.58^a^	-0.42^b^	-0.61^a^	-0.48^b^
IL-8 (pg/mL)	-0.42^b^	-0.54^b^	-0.46^b^	-0.56^b^
IL-18 (pg/mL)	-0.68^a^	-0.53^b^	-0.71^a^	-0.58^b^
IL-1*β* (pg/mL)	-0.50^a^	-0.56^a^	-0.58^a^	-0.61^a^
Adropin (ng/mL)	0.65^a^	0.51^a^	0.78^a^	0.75^a^
Irisin (ng/mL)	0.61^a^	0.52^a^	0.71^a^	0.82^a^
Interpretation of the correlation coefficient (*R*)	Moderate *R*	Moderate *R*	Moderate-strong *R*	Moderate-strong *R*

Data are presented as Pearson's (*R*) coefficients adjusting for variables identified as cofounders in univariate analyses. Moderate correlation coefficient (0.40-0.69); strong correlation coefficient (0.70-0.89). Significance at *p* < 0.05. ^a^*p* < 0.01; ^b^*p* < 0.001. HbA1c: glycated hemoglobin A1c; OPN: osteopontin; CRP: C-reactive protein; TNF-*α*: tumor necrosis factor-*α*. Interleukins (IL-6, IL-8, IL-18, and IL-1*β*).

**Table 6:** Receiver operating characteristic curve analysis of adropin, irisin, osteopontin, miR-146a, and miR-144 for predicting diabetes complications in moderate glycemic controlled type 2 diabetes (>7-8% HbA1c, no = 30).

Variable	AUC	SE	CI (95%)	*p* value	Sensitivity	Specificity
miR-146a	0.81	0.47	0.72-0.98	0.001	91.5%	89.4%
miR-144	0.78	0.51	0.69-0.86	0.01	84.7%	86.8%
Adropin	0.82	0.46	0.73-0.93	0.001	79.1%	71.6%
Irisin	0.78	0.43	0.65-0.89	0.01	76.2%	72.3%
Osteopontin	0.91	0.42	0.75-0.95	0.001	88.6%	75.1%

AUC: area under the curve; SE: standard error; CI: confidence interval.

**Table 7:** Receiver operating characteristic curve analysis of adropin, irisin, osteopontin, miR-146a, and miR-144 for predicting diabetes complications in poor glycemic controlled type 2 diabetes (>8% HbA1c, no = 50).

Variable	AUC	SE	CI (95%)	*p* value	Sensitivity	Specificity
miR-146a	0.91	0.51	0.72-0.98	0.001	94.5%	91.4%
miR-144	0.86	0.53	0.69-0.93	0.001	88.7%	87.8%
Adropin	0.87	0.48	0.73-0.93	0.001	82.1%	79.6%
Irisin	0.82	0.45	0.65-0.91	0.001	79.4%	76.5%
Osteopontin	0.96	0.55	0.75-0.98	0.001	89.7%	79.3%

AUC: area under the curve; SE: standard error; CI: confidence interval.

## Data Availability

All data generated or analyzed during this study are presented in the manuscript. Please contact the corresponding author for access to data presented in this study.
